# Blocking mu-opioid receptors inhibits social bonding in rituals

**DOI:** 10.1098/rsbl.2020.0485

**Published:** 2020-10-14

**Authors:** S. J. Charles, M. Farias, V. van Mulukom, A. Saraswati, S. Dein, F. Watts, R. I. M. Dunbar

**Affiliations:** 1 Brain, Belief, and Behaviour lab; Centre for Trust, Peace and Social Relations, Coventry University, Cheetah Road, Coventry CV1 2TL, UK; 2 Traditional Yoga Association, Reading RG30 3DW, UK; 3 Department of Mental Health, Queen Mary University of London, London E1 4NS, UK; 4 International Society for Science and Religion, Cambridge, UK; 5 Department of Experimental Psychology, University of Oxford, UK

**Keywords:** mu-opioids, social bonding, naltrexone, ritual, ritual bonding

## Abstract

Religious rituals are universal human practices that play a seminal role in community bonding. In two experiments, we tested the role of mu-opioids as the active factor fostering social bonding. We used a mu-opioid blocker (naltrexone) in two double-blind studies of rituals from different religious traditions. We found the same effect across both studies, with naltrexone leading to significantly lower social bonding compared with placebo. These studies suggest that mu-opioids play a significant role in experiences of social bonding within ritual contexts.

## 1. Introduction

There is growing behavioural, physiological and genetic evidence that social bonding, in primates and humans, is underpinned by the mu-opioid endorphin system [1–4]. In humans, the same mechanism seems to underpin both dyadic bonding and group bonding. Religious rituals have long been suggested to play a significant role in community bonding [5], and a number of studies indicate that ritual participants often feel a strong connection with others [6–8]. However, so far, no studies have investigated the pharmacological mechanisms involved.

Rituals often contain many components that are known to release mu-opioids, such as synchronized movement [9], music making [10,11] and/or pain [8,12,13]. These components are also known to foster social bonding [14–17]. We have shown, in a series of large-scale cross-cultural studies of church and yoga groups [18], that religious rituals raise pain thresholds and enhance a sense of social bonding. While providing prima facie evidence of a role for mu-opioids, the hypothesis that these effects explicitly involve mu-opioids has yet to be tested directly.

Since mu-opioids do not pass the blood–brain barrier [19], we use the opioid antagonist naltrexone, which has a preferential binding for mu-receptors [20,21], in a reverse-cause design. Although other studies have used naltrexone in such a design before [22–25], none has investigated the specific context of religious ritual. Indeed, Inagaki [26] has emphasized that more research is needed to study the direct role of opioids in social bonding *during interaction* with others.

To test whether the opioid system is crucially involved in the bonding that takes place during rituals, we conducted two double-blind studies. To ensure that any results were not specific to a particular religious context, study 1 exploited a small-scale laboratory study of yoga classes in the United Kingdom, while study 2 used a larger field study of an Afro-Brazilian Umbanda ritual. Our hypothesis was that, if mu-opioids play a significant role in social bonding during rituals, participants taking a mu-opioid blocker will, compared with those taking a placebo, experience a reduced sense of bonding.

## 2. Study 1: social bonding during yoga

Yoga is a form of structured exercise with religious overtones that satisfies the definition of a religious ritual (see [27]). There is some research linking yoga with the release of β-endorphin [28,29]. We recruited a subset of participants from a five-week laboratory-based study of yoga who agreed to take part in an additional session involving the administration of naltrexone.

### (a) Methods

Ten participants (nine female) agreed to take part in this study. One participant (male) had an adverse reaction to naltrexone and withdrew. In total, nine participants were included (*M*_age_ = 25.8, s.d._age_ = 11.7, all female). Five participants (*M*_age_ = 28.0, s.d._age_ = 15.9) were randomly allocated to the placebo group and four (*M*_age_ = 23.0, s.d._age_ = 2.9) to the naltrexone group. Allocations were made by an author not involved in data collection and stored in an encrypted data file only accessible at the end of the experiment. For the full recruitment and screening procedure, see the pre-registration (https://osf.io/7gn3j/). Exclusion criteria applied in selecting participants are listed in the electronic supplementary material (https://osf.io/y4gw7/). All participants were of European background/ethnicity.

The measure of social bonding consisted of six items, adapted from previous work on social bonding, each measured on a scale of 1 (low) to 7 (high). Five items were verbal: ‘At this moment, how connected do you feel to the people in the group?' [30]; ‘How much do you like the people in the group overall?' [31]; ‘At this moment, how emotionally close do you feel to the other members of this group as a whole?' [32]; ‘Thinking about everyone in this session now, do you feel you have a lot in common with others?' [33]; ‘Thinking about everyone in this session now, how much do you trust the others in this group?'; with one pictorial item (the Inclusion of Others in Self scale, IOS; [34]). Mean response across the six questions was taken as the overall social bonding score. This social bonding scale has been used in previous work and is described in full detail in [18]. Reliability is presented in the results.

As a fast, short-term effect was required [35], we followed [25] and used an oral administration of 100 mg of naltrexone; this dose produces few if any side effects in healthy volunteers [35,36]. Participants were made aware ahead of time of the drug that they could be given, and a procedure was in place in the event of adverse effects.

Hatha Yoga (often dubbed simply ‘yoga') is a physical practice of Indian spiritual origins where participants adopt multiple postures (known as *asanas*). The yoga session was designed by a professional instructor (AS) and can be found at https://osf.io/pxjwd/. The yoga session for this study was the sixth consecutive week of yoga that these participants took part in, allowing individuals to develop a sense of familiarity through repeated exposure/practice in order to fulfil the operational definition of ritual [27].

Participants arrived at the laboratory 1 h prior to the yoga session. Each participant was given a pill bottle that contained two pills of either 2 × 50 mg of naltrexone or the placebo. After taking the pills, they answered a short questionnaire, which included the social bonding scale. They were then given distraction reading material for a 60 min waiting time to allow the naltrexone to become active, after which the yoga session commenced. After a 1 h yoga class, participants completed the post-session questionnaires and were debriefed.

### (b) Results and discussion

The pre-yoga social bonding measure had a McDonald's total omega value of *ω* = 0.87, 95% CI [0.76, 0.98] and the post-yoga questions had *ω* = 0.86, 95% CI [0.70, >0.99], indicating moderate-to-high internal reliability, comparable to previous studies [18].

Shapiro–Wilk tests showed that all social bonding scores for both naltrexone (pre-yoga: *W* = 849, *p* = 0.224; post-yoga: *W* = 950, *p* = 0.714) and placebo (pre-yoga: *W* = 0.911, *p* = 0.475; post-yoga: *W* = 988, *p* = 0.971) were not significantly different from normally distributed and the homogeneity of variances assumption was not violated. Even so, owing to the small sample, it is possible that the parametric assumptions were violated without being detected. Consequently, we used non-parametric analyses.

**Figure 1.**
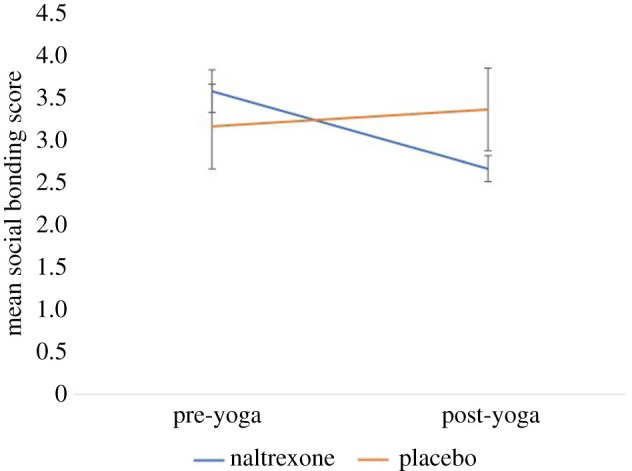
The effect of naltrexone versus placebo on social bonding before and after a yoga session. Error bars represent ±1 s.e.

We used the nparLD package in R to run a non-parametric within–between ANOVA via the f1.ld.f1 function; this produces an ANVOA-like statistic but treats the denominator degrees of freedom as infinite [37]. There was no significant main effect of pill type (*F*_1,∞_ = 0.07, *p* = 0.943) or time (*F*_1,∞_ = 2.34, *p* = 0.071), but there was a significant interaction effect (*F*_1,∞_ = 4.05, *p* = 0.012). Note that effect sizes cannot be directly calculated using the non-parametric within–between ANOVA, but Feys [38] suggests an indirect method for interaction effect sizes. Here, we found the interaction effect size to be *d* = 0.77. This interaction effect means participants who took naltrexone had significantly lower social bonding scores after the ritual than those who took placebo, when compared with before the yoga session (figure 1). The analysis here differs from the pre-registered plan owing to the realization that the original analysis plan was not the most appropriate for the study design. We have, nonetheless, completed the pre-registered analysis, in the analysis script provided. The results in the pre-registered plan were also significant, supporting our hypothesis.

These results provide the first evidence for the role of mu-opioids in creating the sense of bonding associated with religious rituals, though with a small sample. To provide corroboration and to establish the wider cultural generality of this effect [39], we undertook study 2 as part of a larger-scale field study.

## 3. Study 2: social bonding in an Afro-Brazilian religious ritual

Study 2 was conducted during an Umbanda ritual in Brazil. Umbanda is an Afro-Brazilian religion that blends spiritualism, African ritual dances and rhythms, and Roman Catholic prayers and images (see electronic supplementary material for a fuller description: https://osf.io/9r2jb. The full ritual lasted 2 h.

### (a) Methods

Participants were recruited from an Umbanda ritual in Sao Paulo, Brazil that had been part of a larger study of 18 different churches [18]. Twenty-four participants (*M*_age_ = 42.7, s.d._age_ = 15.3, 16 females) who did not meet any exclusion criteria (see electronic supplementary material, https://osf.io/y4gw7/) completed a short questionnaire which included a measure of social bonding. Of these, 11 participants (*M*_age_ = 38.8, s.d._age_ = 13.6, 6 female) were randomly allocated to the naltrexone group and 13 participants (*M*_age_ = 47.7, s.d._age_ = 15.3, 10 female) were randomly allocated to the placebo group.

We used the same measures of social bonding and the same amount of naltrexone (100 mg) as for study 1.

Religious group members were informed in advance of when data collection would be taking place. They attended the religious ritual as usual. Those who consented to take part were first given a medical screening questionnaire. If the participant did not meet any exclusion criteria, they were then provided with the pills. After taking the pills, they completed the pre-ritual questionnaire, attended the ritual and filled out the post-ritual questionnaire before being debriefed.

We conducted a power analysis using a within–between ANOVA instead of a between-participants *t*-test (as in the pre-registration). Using G*Power to calculate the minimum number of participants needed to have an appropriately powered within–between ANOVA with an effect size of *f* = 0.295, *α* = 0.05, power of 0.8, two groups, two measurements and with a sphericity correction of 1. The correlation among repeated measures, calculated using the current study's data, was *r* = 0.788. Using these values, an ANOVA with 12 total participants (six in each condition) would be appropriately powered to find an interaction. Thus, the sample size of 24 participants that we used is more than satisfactory.

### (b) Results

Internal reliability was checked on the social bonding score for both pre- and post-ritual measures, with a pre-ritual McDonald's total *ω* = 0.86, 95% CI [0.72, 0.99] and a post-ritual McDonald's total *ω* = 0.90, 95% CI [0.84, 0.95], which falls within the range of what is considered good reliability.

We used Shapiro–Wilk tests to check whether the data met the assumptions for parametric testing. Although social bonding (pre- and post-service) scores did not differ significantly from normality for either the naltrexone or placebo conditions, the post-service social bonding scores for placebo participants did (*W* = 0.793, *p* = 0.006). Therefore, a non-parametric form of ANOVA was used for the analysis.

**Figure 2.**
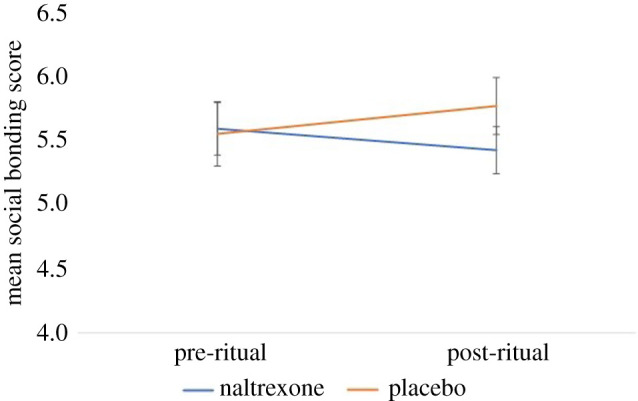
The effect of naltrexone versus placebo on social bonding before and after a religious ritual. There was a significant interaction between pill type and time of measurement. Error bars represent ±1 s.e.

We used the nparLD package in R to run a non-parametric within–between ANOVA via the f1.ld.f1 function; this produces an ANVOA-like statistic but treats the denominator degrees of freedom as infinite [37]. There was no significant main effect of either pill type (*F*_1,∞_ = 0.60, *p* = 0.440) or measurement occasion (*F*_1,∞_ = 0.22, *p* = 0.640), but there was a significant interaction effect (*F*_1,∞_ = 5.28, *p* = 0.022), indicating, when compared with before the ritual, participants who took naltrexone had significantly lower social bonding scores after the ritual than those who took placebo (figure 2). Note that effect sizes cannot be directly calculated using the non-parametric within–between ANOVA, but Feys [38] suggests an indirect method for interaction effect sizes. Here, we found the interaction effect size to be *d* = 0.64 (see analysis script https://osf.io/dw98k/ lines 371–407 for more detail).

This result confirms the findings from study 1 and, importantly, does so with a larger sample and in a very different religious and cultural context. This suggests that the endorphin effect is independent of cultural context.

## 4. General discussion

Previous work on the role of opioids on social bonding has been conducted either via proxy measures [18,40,41] or via daily self-reporting of social bonding after it has taken place [24]. Here, we sought to understand the role of opioids on social bonding in an ecologically valid setting [2,18,42]. We have demonstrated that mu-opioids play a key role in the social bonding experience during ritual by showing that naltrexone, compared with placebo, lowers feelings of bonding. These results were consistent and individually significant across the two studies. This is the first set of studies to demonstrate the causal role of mu-opioids on bonding during a ritual, and we do so in both a laboratory and a field setting.

It has often been suggested that one of the primary functions of religion is to promote social bonding and thus enhance group solidarity (e.g. [43]). These results extend previous work by providing evidence for a mechanism for how group solidarity might be promoted. In so doing, the results support the brain-opioid theory of social attachment [2,44], which argues that the endogenous opioid system is a major neuroendocrine system underlying social bonding.

Although the sample size of study 1 is small, it adds significantly to study 2 by showing that the results hold across two different cultures and ritual types, thereby providing strong ecological validity [39]. Although it is possible that other neurochemicals such as oxytocin [45,46] and dopamine [47] might also play a role in the social bonding experience, studies of the receptor genetics for these other neurochemicals suggest that these play a more specialized and much less prominent role compared with β-endorphins [1,4]. Still, future research could seek to rule out the role of other such neurochemicals that have been proposed to play a role in bonding in further double-blind studies to determine which neurochemicals are necessary and/or sufficient for social bonding to occur. Study 1 (but not study 2) suffered from the limitation that it recruited very few males, and it would be desirable to increase the gender representation in future studies. It should also be noted that naltrexone may also block the kappa-opioid receptors [20,21], which have a particular affinity with dynorphins. Although this makes it difficult to be absolutely certain that the primary target is the mu-receptors, primate social bonding has been explicitly identified in previous studies with the β-endorphins [48], which have a particular affinity for the mu-receptors.

In summary, we provide the first placebo-controlled, double-blind studies to examine the pharmacological basis for the role of religious rituals in social bonding. These studies provide a prima facie case on the neurochemical mechanisms underlying ritual social bonding.

## Supplementary Material

Supplementary image

## Authors' note

A link to the pre-registrations, procedures, protocols, data and analysis scripts can be found on the Open Science Framework's online repository for the respective studies: UK study, https://osf.io/z87p4/; Brazil study, https://osf.io/4kpb6/. A pre-print has also been made available and is accessible at: https://psyarxiv.com/sdrp4/.

## Data accessibility

The data for this project can be found both in the associated OSF projects, linked below and on the Dryad Digital Repository [49]: UK study: https://osf.io/z87p4/ Brazil study: https://osf.io/4kpb6/.
