# Supplementary figures and images for: Blocking mu-opioid receptors inhibits social bonding in rituals
Source: Biol Lett. 2020 Oct 14;16(10):20200485. doi: 10.1098/rsbl.2020.0485 (PMC7655482; doi:10.1098/rsbl.2020.0485)

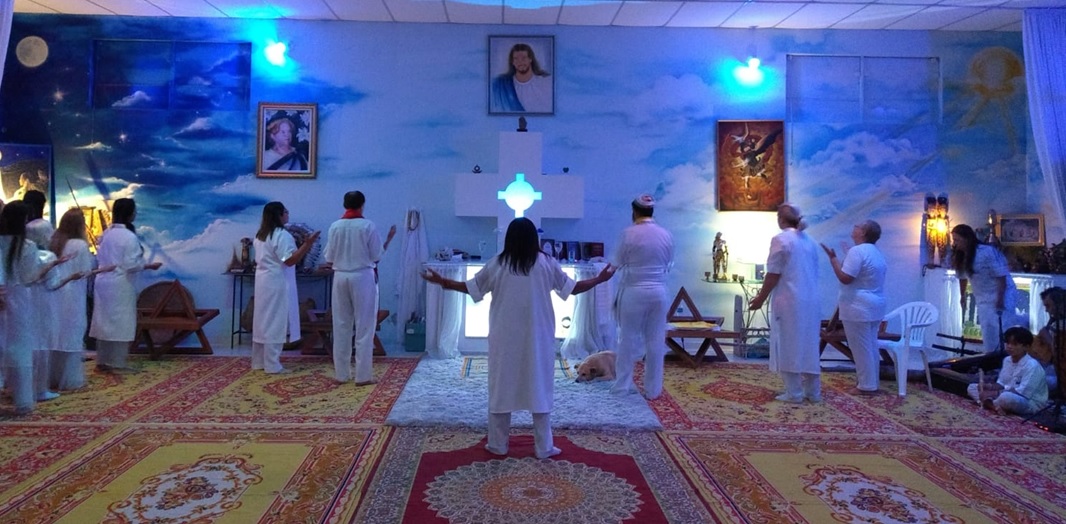

Supplement: Supplementary image [file rsbl20200485supp1.jpg]
